# Antifungal Efficacy during *Candida krusei* Infection in Non-Conventional Models Correlates with the Yeast *In Vitro* Susceptibility Profile

**DOI:** 10.1371/journal.pone.0060047

**Published:** 2013-03-28

**Authors:** Liliana Scorzoni, Maria Pilar de Lucas, Ana Cecilia Mesa-Arango, Ana Marisa Fusco-Almeida, Encarnación Lozano, Manuel Cuenca-Estrella, Maria Jose Mendes-Giannini, Oscar Zaragoza

**Affiliations:** 1 Mycology Reference Laboratory, National Centre for Microbiology, Instituto de Salud Carlos III, Madrid, Spain; 2 Laboratório de Micologia Clínica, Faculdade de Ciências Farmacêuticas, Universidade Estadual Paulista de São Paulo, Araraquara, Brazil; 3 Department of Cellular Biology, National Centre for Microbiology, Instituto de Salud Carlos III, Madrid, Spain; 4 Group of Investigative Dermatology, University of Antioquia, Medellín, Colombia; Universidade de Sao Paulo, Brazil

## Abstract

The incidence of opportunistic fungal infections has increased in recent decades due to the growing proportion of immunocompromised patients in our society. *Candida krusei* has been described as a causative agent of disseminated fungal infections in susceptible patients. Although its prevalence remains low among yeast infections (2–5%), its intrinsic resistance to fluconazole makes this yeast important from epidemiologic aspects. Non mammalian organisms are feasible models to study fungal virulence and drug efficacy. In this work we have used the lepidopteran *Galleria mellonella* and the nematode *Caenorhabditis elegans* as models to assess antifungal efficacy during infection by *C. krusei*. This yeast killed *G. mellonella* at 25, 30 and 37°C and reduced haemocytic density. Infected larvae melanized in a dose-dependent manner. Fluconazole did not protect against *C. krusei* infection, in contrast to amphotericin B, voriconazole or caspofungin. However, the doses of these antifungals required to obtain larvae protection were always higher during *C. krusei* infection than during *C. albicans* infection. Similar results were found in the model host *C. elegans*. Our work demonstrates that non mammalian models are useful tools to investigate *in vivo* antifungal efficacy and virulence of *C. krusei*.

## Introduction

Fungal infections have emerged worldwide due to a growing population of immunosuppressed patients, including patients with cancer, AIDS, solid-organ and hematopoietic stem cell transplant recipients, premature neonates, and patients recovering from major surgery [1]–[5]. These infections have significant morbidity and mortality rates and are difficult to prevent, diagnose and treat [6]–[8].

*Candida* spp are commensal yeasts responsible for different clinical manifestations, from mucocutaneous overgrowth to blood stream infections [1], [9]–[12]. *Candida albicans* is still the major cause of invasive fungal disease. However, a growing number of infections produced by non-*albicans Candida* spp has been reported in the last years [1], [13]–[15]. Among them, there are some species that are intrinsically resistant or have reduced susceptibility to antifungals. The massive use of antifungals in prophylaxis, such as fluconazole, has facilitated the selection of pathogenic fungi resistant to these agents [16]–[19].

*Candida krusei* is an opportunistic pathogen which presents intrinsic resistance to fluconazole. The infection is associated with the prophylactic or therapeutic use of this antifungal agent [20]–[23]. Two mechanisms of azole resistance in *C. krusei* have been described: overexpression of drug efflux pumps [24] and diminished sensitivity of the target enzyme, the cytochrome P450 sterol 14-demethylase (encoded by the *CYP51* gene) [25]. Diseases caused by *C. krusei* have high associated mortality (30–60%) [26], [27]. Despite the intrinsic resistance to fluconazole, *C. krusei* is usually susceptible to voriconazole *in vitro*, which correlates with the binding of this drug to the target enzyme [21].

Antifungal resistance *in vitro* does not always correlate with clinical resistance. The best correlation between *in vitro* and clinical efficacy is found in HIV-positive patients with oropharyngeal candidiasis [22], [28], [29]. In contrast, although *C. parapsilosis* shows reduced *in vitro* susceptibility to echinocandins, these antifungals have been shown to be effective in the treatment of invasive candidiasis caused by this species [30].

The use of invertebrate hosts has recently emerged and facilitated the study of fungal pathogenesis. Among these non-mammalian hosts, amoebae (*Acanthamoeba castellanii*, *Dictyostellium discoideum*), nematodes (*Caenorhabditis elegans*) and insects (*Drosophila melanogaster*, *Galleria mellonella*) have been successfully used to study the virulence of some fungi [31]–[35]. Moreover, some aspects of the innate response are conserved between these hosts and mammals [36]. *Galleria mellonella* is a lepidopteran (Pyralidae) that provides important advantages as host model. The larvae can be incubated in a range of temperature between 25 to 37°C, so it is possible to simulate the natural fungal habitat and the infection conditions in mammals. In addition, as in mammalian models, it is possible to introduce by injection exact doses of pathogens to the larvae, which poses a technical improvement over other non-conventional hosts. *Galleria mellonella* has six types of phagocytic cells that play an important role in the defense system [37], [38]. The density of these cells in the haemolymph is not constant, and changes during infection can be easily measured and used as a parameter of the response of the larvae after exposure to pathogens [39]. The viability of the larvae can be easily recorded by the lack of movement and the massive melanization induced by *G. mellonella* in response to infection [40]–[42]. Another organism that is used as model host is the soil nematode *Caenorhabditis elegans*, which feeds on microorganisms, but is susceptible to bacterial and fungal pathogens [33], [43]–[45]. *Caenorhabditis elegans* has been used to study virulence, filamentation and antifungal efficacy of antifungal drugs [44], [46].

In this study, we initially aimed to characterize the interaction between *G. mellonella* and *C. krusei* with two purposes: 1) To get insights about virulence traits of this pathogenic yeast, and 2) to investigate if antifungal efficacy *in vivo* correlates with the susceptibility profile shown by *C. krusei in vitro*. Furthermore, we have complemented our studies with *C. elegans*, and observed similar behaviors, indicating that non-conventional models can be used to investigate *C. krusei* virulence and antifungal efficacy.

## Materials and Methods

### Strains and media

*Candida albicans* SC5314 [47], *C. krusei* ATCC 6258 and two clinical isolates (CL8053 and CL80317) from the Yeast Collection of the Mycology Reference Laboratory of the Spanish National Centre for Microbiology and *Cryptococcus neoformans* variety *grubii* (H99 strain, ATCC 20882) were used in this study. The yeasts were grown overnight in liquid Sabouraud medium (Difco, BD, USA) at 30°C with shaking. *Escherichia coli* OP50 strain was obtained from the *Caenorhabditis* Genetics Center (University of Minnesota) and was maintained on LB agar plates at 37°C.

### Antifungal susceptibility testing (AFST)

Minimum inhibitory concentration (MIC) values were determined using the EUCAST protocol [48], [49]. For AFST, 10 different clinical isolates of *C. albicans* and 10 clinical isolates of *C. krusei* were obtained from the yeast collection of the Mycology Reference Laboratory of the Spanish National Centre for Microbiology. Data were expressed as geometric mean, mode, range (minimum-maximum) and MIC frequency distribution.

### Insect larvae manipulation and incubation conditions

*Galleria mellonella* larvae (0.3–0.5 g, R.J. Mous Livebait, The Netherlands) were placed in Petri dishes and incubated at 37°C in the dark the night before the experiments. Larvae with color alterations (i.e. dark spots or with apparent melanization) were excluded. Antifungals and yeast suspensions were injected in the haemocele through the last left pro-leg of the larvae using a 10 µL Hamilton syringe (Hamiltion, USA). The pro-leg had been previously cleaned with 70% ethanol. A total of 10 µL were injected in each larva. Larvae death was monitored by visual inspection of the color (brown-dark brown) and by lack of movement after touching them with forceps. For each condition, a total of 20 larvae were used, and each experiment was repeated at least twice. After infection, larvae were incubated at 25, 30 or 37°C.

### Survival assay

Yeasts were grown overnight in liquid Sabouraud, washed with PBS, and suspended in the same buffer. Cell density was estimated with an Automatic Cell Counter TC10 (Bio Rad). For survival assays, larvae were inoculated with 10^7^, 5×10^6^ and 2.5×10^6^ cells/larva of *C. krusei* and 10^6^, 5×10^5^ and 10^5^ cells/larva of *C. albicans*. The inocula were prepared in PBS plus 20 mg/L of ampicillin to prevent bacterial contamination. The infected larvae were incubated at 25°C, 30 or 37°C, and the death was daily monitored during 7 days.

### Growth curve at different temperatures

Yeast strains were grown overnight and diluted in fresh Sabouraud liquid medium at 10^3^ cells/mL. Two hundred microliters of this suspension were placed in 96-well microdilution plates, and incubated at 25, 30 or 37°C in a Labsystems IEMS Reader MF spectrophotometer. Optical density (OD) was determined at 530 nm every hour during 72 hrs.

### *In vivo* phagocytosis assay

Yeast cells were stained with 10 µg/mL Calcofluor white (Sigma, St. Louis, MO) for 30 min at 37°C. Then, these cells were injected into *G. mellonella* larvae (10^7^ cells/larva, 5 per group), and phagocytosis was analyzed after 3 hrs of incubation at 25 and 37°C. Haemolymph was collected in 1.5 mL tubes and diluted 1∶1 in IPS buffer (Insect Physiological Saline: 150 mM sodium chloride, 5 mM potassium chloride, 10 mM Tris-HCl pH 6.9, 10 mM EDTA and 30 mM sodium citrate) to avoid coagulation and melanization of the haemolymph. Haemocytes were placed on a slide and phagocytosis was visually quantified using a Leica DMI 3000B microscope. One hundred haemocytes from each larva were counted in each case, and the percentage of haemocytes containing yeasts was calculated and plotted. *Cryptococcus neoformans* H99 strain was used as control. Phagocytosis was also analyzed in larvae infected in the same way, but treated with 64 mg/kg fluconazole or 4 mg/kg amphotericin B.

### Determination of haemocyte density

Groups of five *G. mellonella* were infected with 10^7^ yeast cells/larvae and incubated at 37°C for 3 hrs. The haemolymph of each larva was collected in 1.5 mL tubes and diluted 1∶10 in IPS buffer. The cells were counted using a haemocytometer.

### Measurement of in vivo filament formation

*Galleria mellonella* was infected with 10^7^ cells/larva of *C. albicans* and *C. krusei* strains. The larvae were incubated at 37°C for 24 hours. Larvae were macerated in 100 µm nylon cell strainers (Falcon, BD, USA) with 1 mL of IPS. The liquid was then collected, centrifuged and suspended in 1 mL of the same buffer. Samples were stained with Calcofluor white (Sigma, St. Louis, MO), as described above, and yeast morphology was observed with a Leica DMI 3000B fluorescence microscope.

### Melanization quantification

Larvae were infected with PBS, 5×10^5^, 10^6^ and 5×10^6^ cells/larva of *C. krusei*. Then, the haemolymph of each larva was collected after 3 and 24 hrs in 1.5 mL tubes and diluted 1∶10 with IPS buffer. The samples were placed in 96 well microdilution plates. To quantify melanin levels, we took advantage of existing protocols that quantify laccase activity by detecting the final product of the reaction by measuring the OD in the visible range (400–500) [50], [51]. In our conditions, we observed that 405 nm was an optimal OD to quantify larval melanin and to correlate the results with the visualization of the dark compound. So the OD at 405 nm was measured using a Labsystems IEMS Reader MF spectophotometer. Melanization of larvae infected with *C. albicans* (5×10^5^ cells/larva) and *C. krusei* (5×10^6^ cells/larva) and treated with 64 mg/kg fluconazole and 4 mg/kg amphotericin B was also evaluated.

### Treatment with antifungal drugs

Infected larvae were treated with amphotericin B (1, 2 or 4 mg/kg, Sigma Aldrich Quimica, Madrid, Spain), fluconazole (128, 64, 32, 12, or 4 mg/kg, Pfizer SA, Madrid, spain), voriconazole (7.5 or 10 mg/kg, Pfizer SA, Madrid, Spain) or caspofungin (1, 2 or 4 mg/kg Merck & Com, Inc, NJ, USA). In some experiments, a combination of fluconazole and amphotericin B was also used. Antifungals were applied immediately after the infection. Groups of 10 larvae were treated with the antifungals alone to test the toxicity.

### Fungal burden determination

Infected larvae were selected at different times of infection, washed with 70% ethanol and cut into small pieces with a scalpel. Two mL of PBS-ampicillin were added and the mix was homogenized gently with a vortex and glass beads for 10 seconds. The mix was finally suspended in 9 mL of PBS-ampicillin. Different dilutions were made for each sample and 50 µL from these dilutions were placed on Sabouraud-cloramphenicol agar plates (Oxoid). The plates were incubated at 37°C for 48 h, and the number of colony forming units (CFUs) was determined.

### Histology

Three larvae from different groups (uninfected, infected and/or treated with antifungals) were collected on different days of the infection. The larvae were preserved in 70% ethanol and longitudinal incisions were made with a scalpel in the dorsal part. The samples were fixed with 10% buffered formaline for 24 hrs. Then, the samples were dehydrated with increasing concentrations of ethanol, rinsed with xylol, and embedded in paraffin. Tissue sections (5 microns) were stained with periodic acid Schiff (PAS) solution and observed with a Leica DMI3000 microscope.

### *Caenorhabditis elegans* strain and infection conditions

The following *C. elegans* mutant strain, obtained from CGC, was used in all experiments: AU37 (*glp-4*(*bn2*) I; *sek-1*(*km4*) X). This strain was grown on agar plates seeded with *E. coli* OP50 and incubated at 15°C according to standard procedures [52]. This strain is usually chosen for virulence and antifungal efficacy assays because *glp*-4 mutants are sterile at 25°C. This allows to easily following up the survival of the individual animals from the beginning to the end of the experiment and avoids mixing with their progeny [33], [44]. The *sek-1* gene encodes a mitogen-activated protein kinase which is important for the defense of *C. elegans* against microbial infections [33], [44]. Therefore worms defective for *sek-1* are more susceptible to infection and die earlier than wild-type *C. elegans* animals. *Candida* strains were cultivated in liquid Sabouraud medium (Difco, BD, USA) at 35°C with shaking. One hundred µL from this culture were inoculated on solid BHI media (Difco) containing kanamycin (90 µg/mL) and ampicillin (200 µg/mL) and incubated at 30°C for 24 hours. Synchronized worms in the L4 stage were added to the center of the agar plates inoculated with the yeast strains lawns and incubated for three hours at 25°C. In parallel, L4 worms were placed on agar plates containing lawns of *E. coli* OP50 strain. After the three hours incubation, worms were washed with M9 and transferred to 12-well plates with 1 mL 60% M9 buffer [45], 40% BHI, 10 µg/mL cholesterol in ethanol, 200 µg/mL ampicilin and 90 µg/mL kanamycin. Around 20–30 worms were placed in each well. For antifungal efficacy, amphotericin B (1 and 2 µg/mL), fluconazole (12 µg/mL), voriconazole (0.25, 7.5 and 10 µg/mL), caspofungin (2, 4 and 6 µg/mL), or a combination of amphotericin B (1 µg/mL) plus fluconazole (12 µg/mL) were added to the media. Plates were incubated at 25°C and individual worm survival was monitored daily. Nematodes were considered dead when they did not respond to touching. A minimum of two independent experiments was carried out for each treatment. Images were captured with a video camera (JVC KY-F550) attached to a dissecting microscope (Leica MZ7.5).

### Statistics

Graphs and Statistics analyzes were performed with Graph Pad Prisma 5 (La Jolla CA, USA). Survival curves were analyzed by Log-rank (Mantel-Cox) Test and phagocytosis assays, haemocyte density, melanization quantification and fungal burden were analyzed using t-Test.

## Results

### *Candida krusei* killed *G. mellonella* in a dose dependant manner

**Figure 1:**
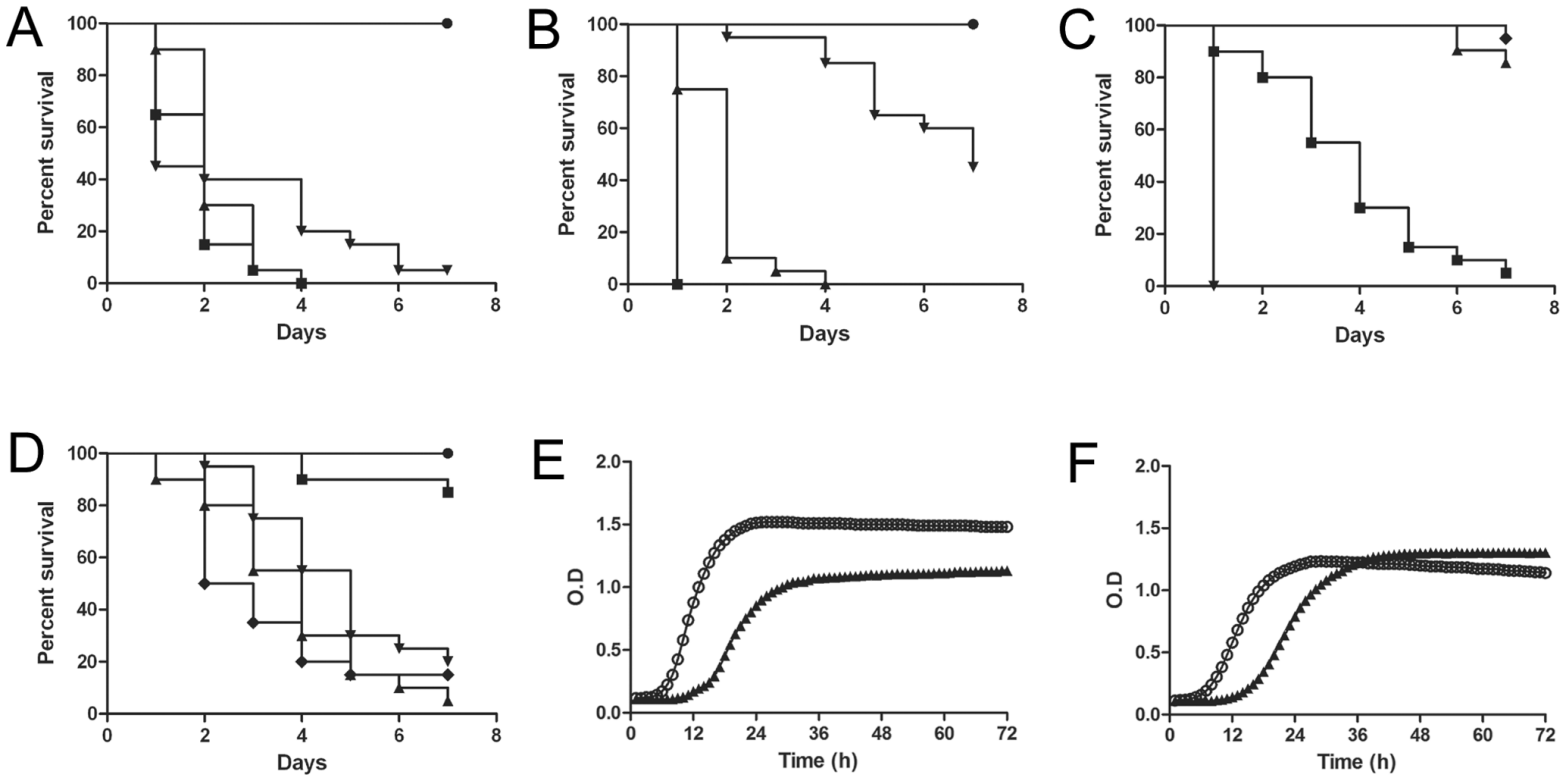
Comparison of the virulence of *C. krusei* and *C. albicans* in *G. mellonella*. (**A**) Survival curve of *G. mellonella* infected with different inocula of *C. krusei* ATCC 6258 • PBS; ▪ 10^7^ cells/larva; ▴ 5×10^6^ cells/larva;▾ 2.5×10^6^ cells/larva incubated at 37°C (**B**). Survival curve of *G. mellonella* infected with different inocula of *C. albicans* SC5314 • PBS; ▪ 10^6^ cells/larva; ▴ 5×10^5^ cells/larva; ▾10^5^ cells/larvae (**C**) Survival of *G. mellonella* infected with inactivated yeast. Control dead cells • PBS; ▪ *C. krusei* ATCC 6258 5×10^6^ cells/larva; ▴ *C. krusei* ATCC 6258 5×10^6^ cells/larva (dead); ▾ *C. albicans* SC5314 10^6^ cells/larva; ♦ *C. albicans* SC5314 10^6^ cells/larva (dead) (**D**); Effect of the incubation temperature on the virulence of *C. albicans* and *C. krusei*. • PBS; ▴ C. *krusei* ATCC 6258 (37°C); ▾C. *krusei* ATCC 6258 (30°C); ♦ *C. albicans* SC5314 (37°C); ▪*C. albicans* SC5314 (30°C); Growth curves of *C. albicans* (E) and *C. krusei* (F) at different temperatures. ○ 37°C; ▴ 30°C.

We first investigated if *C. krusei* killed *G. mellonella*. Our results showed that *G. mellonella* is susceptible to *C. krusei* infection (Figure 1A). The death rate of the larvae depended on the yeast dose injected. Most reproducible results were found when larvae were infected with 5×10^6^ *C. krusei* cells. However, *C. krusei* was less virulent than other fungi, such as *C. albicans*, which killed *G. mellonella* at lower doses (5×10^5^, Figure 1B). To confirm that the death was not a consequence of a shock due to big amounts of yeast injected in the larvae, we inoculated a group of larvae with yeast inactivated by incubation in 4% paraformaldehyde. As shown in Figure 1C, inactivated yeast did not kill *G. mellonella*, confirming that larvae death was dependent on living yeast.

To verify if *C. krusei* virulence in *G. mellonella* depended on the temperature at which the larvae are incubated, we compared virulence of *C. krusei* and *C. albicans* at different temperatures (25, 30 or 37°C). *Candida albicans* was more virulent at 37°C than at 30°C. In contrast, no statistical difference was observed in the survival of *G. mellonella* infected with *C. krusei* and incubated at the different temperatures, indicating that *C. krusei* virulence does not depend on temperature (Figure 1D). Similar findings were obtained with *C. krusei* clinical isolates (result not shown). We also studied the virulence of these two species at environmental temperature (25°C). In agreement with the previous data, we found that *C. krusei* was virulent at 25°C, while *C. albicans* virulence was significantly decreased at this temperature (data not shown). To confirm these results, we investigated if *C. krusei* growth was affected by temperature in a similar manner as *C. albicans*. So we performed growth curves of both species at 30 and 37°C. As shown in Figure 1E and F, *C. albicans* grew better at 37°C compared to 30°C (two-fold reduction in generation time). In contrast, *C. krusei* grew similarly at both temperatures (0.85 fold decrease in generation time when the cells were grown at 37°C compared to 30°C). We found similar results at 25°C (data not shown), supporting that *C. krusei* growth is not affected by the incubation temperature. The final OD reached at the stationary phase at different temperatures was different with both species. *Candida albicans* reached higher OD at 37°C, which differed from the situation found in *C. krusei*, where the final OD at the stationary phase was almost identical at 30 and 37°C. Latency period was longer at 30°C, but the same trend was observed in both species (Figures 1E and 1F).

### Yeast inoculation caused early melanization of the larvae

**Figure 2:**
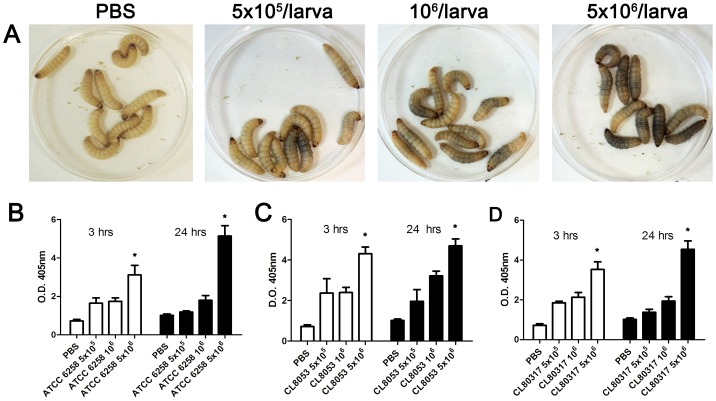
Melanization of *G. mellonella* infected with *C. krusei*. (**A)** Visual appearance of *G. mellonella* larvae infected with different *C. krusei* doses. (B, C and D) Optical Density (OD) of the haemolymph of *G. mellonella* infected with *C. krusei* ATCC 6258 (**B**), clinical isolate CL8053 (**C**) and CL80317 (**D**) with 5×10^5^, 10^6^, 5×10^6^ cells/larva. The different size inoculum reveals dose-response melanization (* p<0.05). All the experiments in this figure were performed at 37°C.

*Galleria mellonella* larvae appeared melanized after a few minutes of *C. krusei* injection (Figure 2A). To quantify this phenomenon, we collected the haemolymph and measured its optical density at 405 nm. When larvae were infected with 5×10^6^ *C. krusei* cells, there was a significant accumulation of melanin in the haemolymph (4.3 times compared to the non-infected larvae), and this melanization increased over time (5 times at 24 hrs, Figure 2B). Clinical isolates showed a similar behavior (Figures 2C and D). We evaluated if *C. krusei* induced melanization of *G. mellonella* at lower temperatures, and we found that this phenomenon also occurred at 25°C (data not shown).

### Phagocytosis and effect of *C. krusei* on haemocyte density

**Figure 3:**
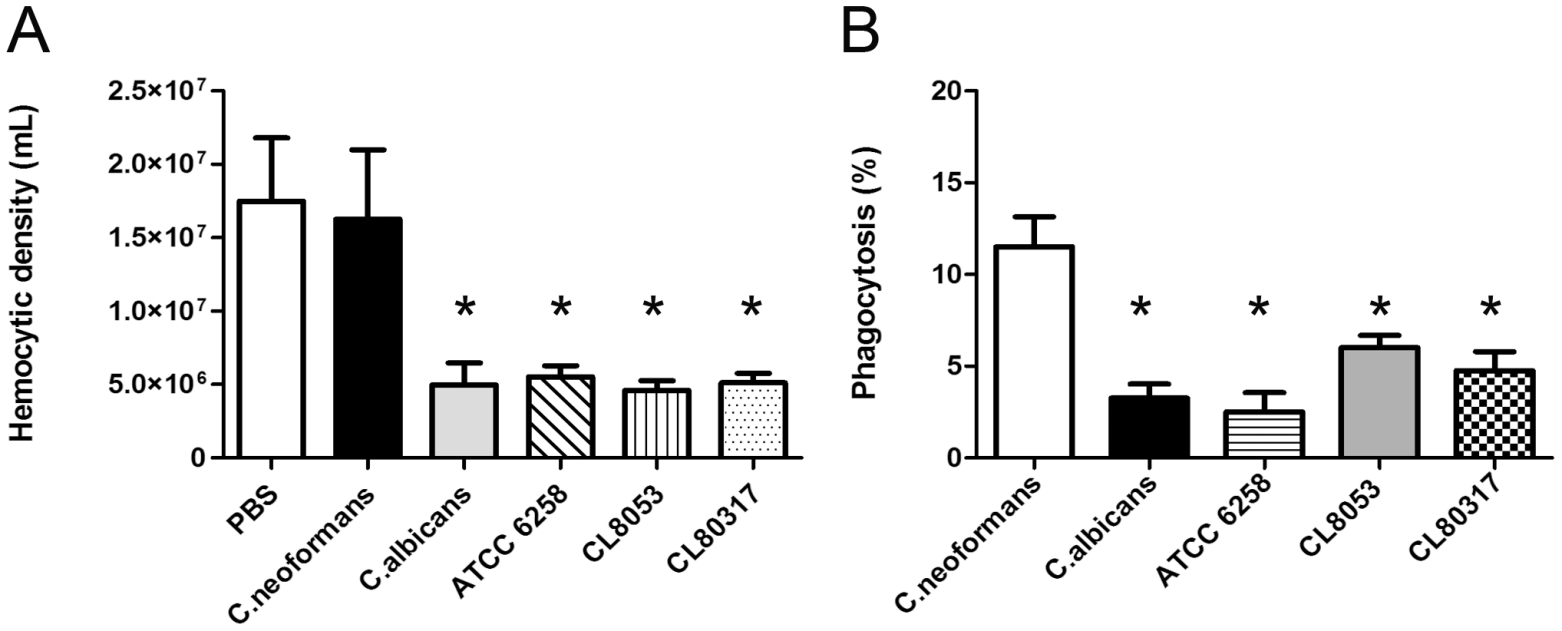
Interaction between *C. krusei* and haemocytes. (**A**) Changes in haemocyte density during *C. krusei* infection. The haemolymph of infected larvae with *C. neoformans, C. albicans* SC5314, *C. krusei* ATCC 6258, CL8053 and CL80317 clinical isolates and PBS was collected and the concentration of haemocytes was estimated using a haemocytometer (**B**). Phagocytosis percentage of *C. neoformans*, *C. albicans* SC5314, *C. krusei* ATCC 6258, CL8053 and CL80317 clinical isolates. Asterisks denote differences statistically significant (p<0.05).

We examined if different *C. krusei* strains had any effect on haemocyte density. As shown in Figure 3A, *C. krusei* produced a decrease in haemocyte density in a similar manner to *C. albicans*.

We then investigated if *C. krusei* cells were phagocytosed by *G. mellonella* haemocytes. We compared the phagocytosis of this pathogen to the one measured with *C. albicans* and *C. neoformans*. The phagocytosis for all *Candida* strains (*albicans* and *krusei*) was significantly lower to the phagocytosis observed with *C. neoformans* (Figure 3B). The same result was found when phagocytosis was assessed at 25°C (data not shown).

*Candida krusei* can filament *in vitro*, so we investigated if this change also took place during infection in *G. mellonella*. We included *C. albicans* in these experiments as control, since it has been reported that this yeast can form hyphae in this model host [53]. As expected, *C. albicans* efficiently produced filaments in the larvae. *Candida krusei* also produced filaments, and in *G. mellonella* crude extracts they were frequently found in clumps of fat body of dark color, which we believe that are composed mainly of insect melanin. This fact may explain the fast melanization of *G. mellonella* when infected with *C. krusei*.

### Antifungal efficacy during *C. krusei* infection in *G. mellonella*

**Figure 4:**
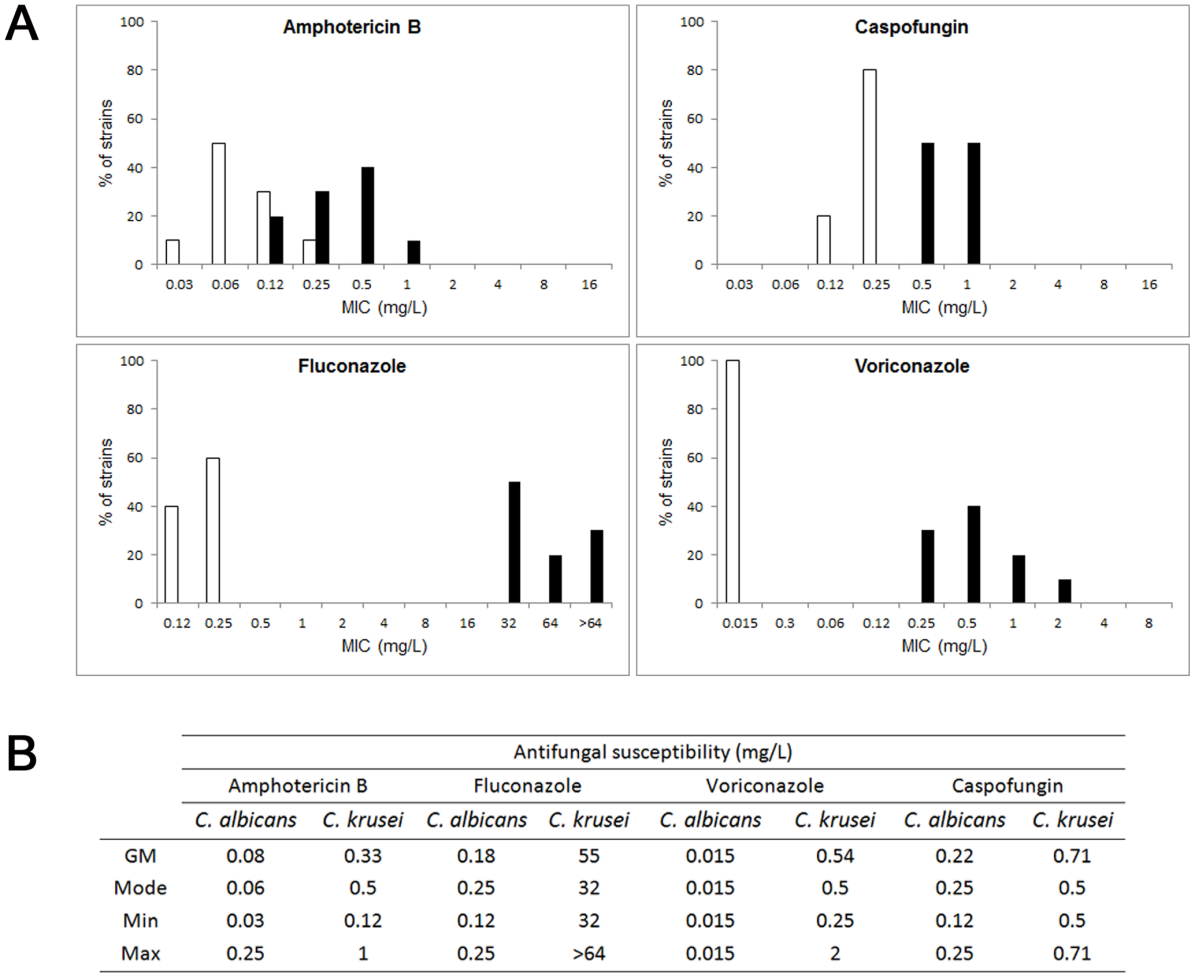
Antifungal susceptibility profile of *C. krusei* and *C. albicans*. A) Distribution of MIC values (n = 10) to amphotericin B, caspofungin, fluconazole and voriconazole of *C. albicans* (white bars) and *C. krusei* (black bars). B) Description of antifungal susceptibility of *C. albicans* and *C. krusei* to different antifungals. N = 10. The geometric mean (GM), mode, minimum (Min) and maximum (Max) are shown.

**Figure 5:**
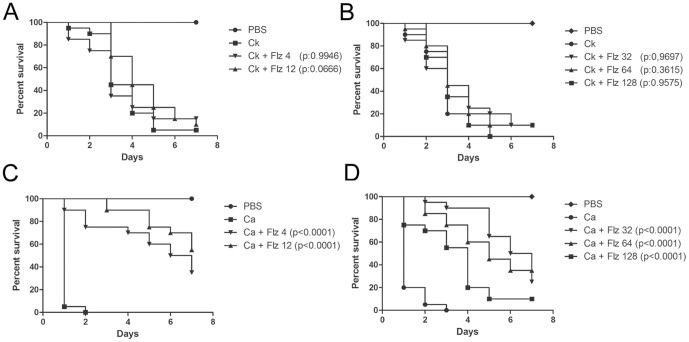
Efficacy of fluconazole during *G. mellonella* infection with *C. krusei* or *C. albicans*. Effect of fluconazole during infection of larvae with 5×10^6^ cells of *C. krusei* (ATCC 6258) per larvae (**A and B**) and 5×10^5^ cells of *C. albicans* cells (SC5314) per larva (**C and D**) in *G. mellonella*. Fluconazole treatment with 4 or 12 mg/kg (**A and C**); 32, 64 or 128 mg/kg (**B and D**).

**Figure 6:**
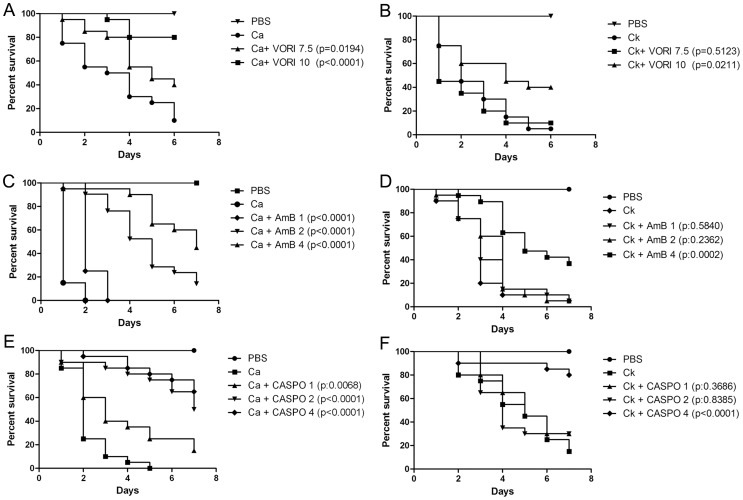
Efficacy of voriconazole, amphotericin B or caspofungin during *C. krusei* and *C. albicans* infection in *G. mellonella*. **A and B**) Voriconazole treatment efficacy (7 and 10 mg/kg) in *G. mellonella* infected with *C. albicans* SC5314 (**A**) or *C. krusei* ATCC 6258 (**B**). **C and D**) Amphotericin B treatment efficacy (1, 2, 4 mg/kg) in *G. mellonella* infected with *C. albicans* SC5314 (**C**) or *C. krusei* ATCC 6258 (**D**). **E and F**) Caspofungin treatment efficacy (1, 2, 4 mg/kg) in *G. mellonella* infected with *C. albicans* SC5314 (**E**) or *Candida krusei* ATCC 6258 (**F**). In all the cases, the larvae were infected with 5×10^5^ *C. albicans* cells/larva and 5×10^6^ *C. krusei* cells/larva.

One of the main features for *C. krusei* is its *in vitro* susceptibility profile. As shown in Figure 4, *C. krusei* is less susceptible to amphotericin B, voriconazole and caspofungin than *C. albicans*, and intrinsically resistant to fluconazole. So we studied if this phenotype correlated with a lack of response to the antifungal during infection in *G. mellonella*. For this purpose, we infected *G. mellonella* with *C. krusei* or *C. albicans*, and treated the larvae with different antifungals (fluconazole, voriconazole, amphotericin B and caspofungin). In the case of larvae infected with *C. krusei*, treatment with fluconazole, even at very high doses (32 or 64 mg/kg) did not increase the survival (Figures 5A and B). At higher concentrations (128 mg/kg), there was a decrease in the survival, which is explained by the toxicity of the antifungal at this high concentration, which induced 25% of death after 7 days of treatment (data not shown). When the same experiments were performed with *C. albicans*, treatment with all the fluconazole concentrations produced significant survival (Figures 5C and 5D). Concerning other azoles, *C. krusei* is considered susceptible to voriconazole, although it presents higher MIC values to this antifungal than *C. albicans* (see Figure 4). So we studied the efficacy of voriconazole during infection in *G. mellonella*. We found that both voriconazole concentrations tested (7.5 and 10 mg/kg) protected larvae from *C. albicans* infection (Figure 6A). In contrast, larvae infected with *C. krusei* were only protected with higher voriconazole concentrations (Figure 6B). Lower doses did not have any effect on survival.

Amphotericin B (4 mg/kg) prolonged survival of larvae infected with *C. albicans* at all the concentrations tested (Figure 6A). In contrast, amphotericin B only protected larvae infected with *C. krusei* at the highest dose (4 mg/kg), which produced a 60% survival at the fourth day (Figure 6B). In a similar way, caspofungin was effective during *C. albicans* infection at all the doses tested (Figure 6C), while it only protected larvae inoculated with *C. krusei* at the highest dose (4 mg/kg) (Figure 6D). We also used an antifungal combination with fluconazole (12 or 4 mg/kg) and amphotericin B at a sub-therapeutic dose in *G. mellonella* (1 mg/kg), but we found no synergic effect between the antifungals (data not shown).

### Fungal burden determination and histopathology

**Figure 7:**
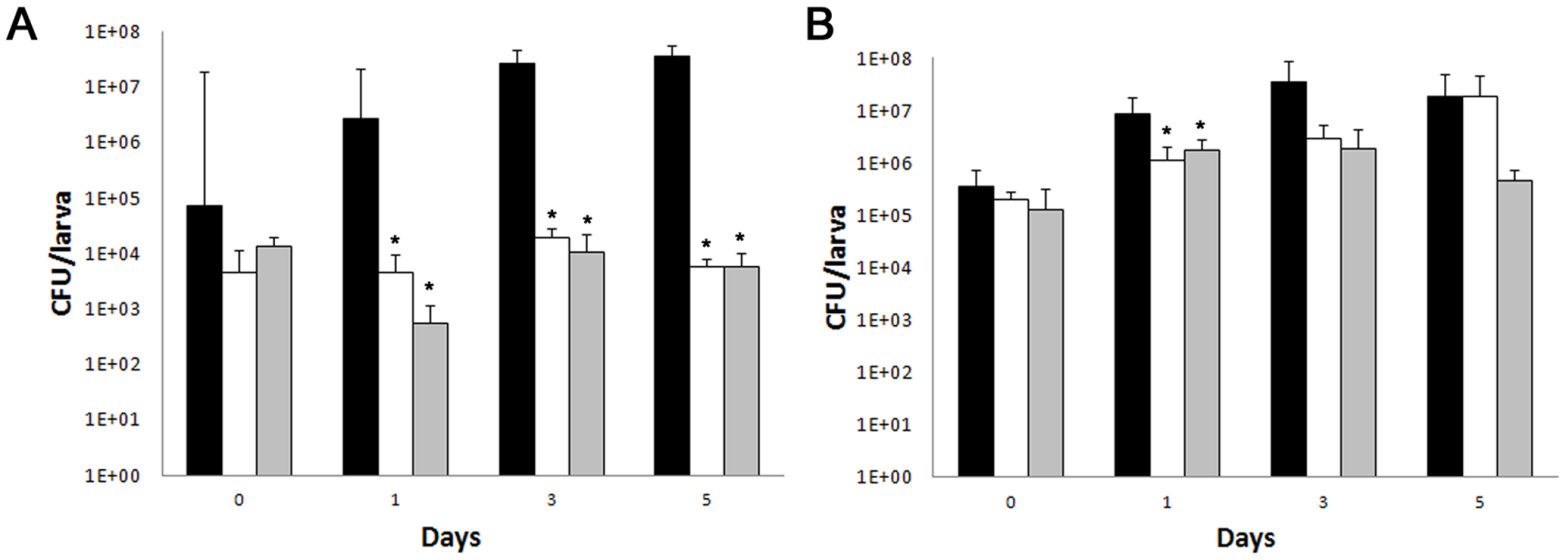
Effect of antifungal treatment on fungal burden in *G. mellonella* infected with *C. albicans* or *C. krusei*. *Galleria mellonella* larvae were infected with *C. krusei* ATCC 6258 (**A**, 5×10^6^ cells/larva) or *C. albicans* SC5314 (**B**, 5×10^5^ cells/larva) and CFUs recovered from *G. mellonella*. Black bars, no treatment, white bars, fluconazole (12 mg/kg), grey bars, amphotericin B (4 mg/kg).

The fungal burden was determined by recovering the yeast cells from the larvae infected with *C. albicans* or *C. krusei* and treated with fluconazole (12 mg/kg) or amphotericin B (4 mg/kg). The number of CFUs increased in larvae infected with both pathogens with the time of infection (Figure 7). Treatment of larvae infected with *C. albicans* with fluconazole or amphotericin B decreased the number of CFUs by 1000-fold (Figure 7A). In larvae infected with *C. krusei*, amphotericin B reduced the fungal burden by 10-fold. Curiously, fluconazole also reduced the initial fungal burden, although it did not have an effect after longer times (5 days, Figure 7B).

**Figure 8:**
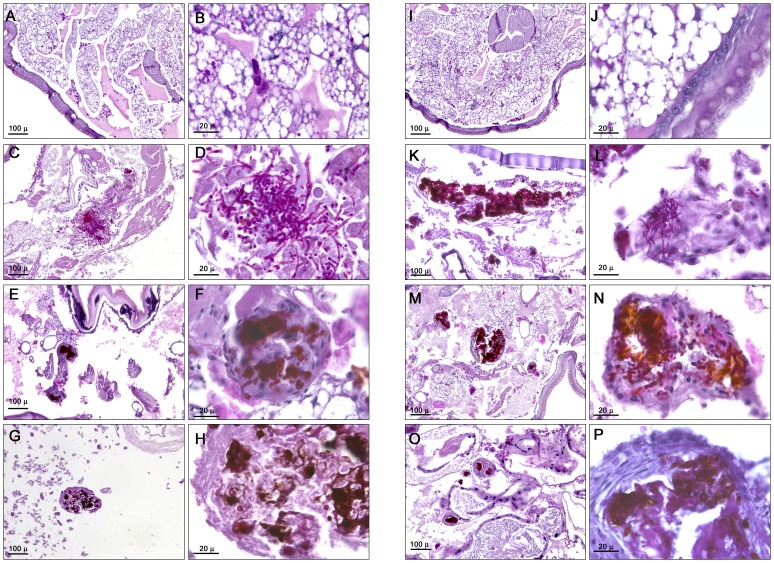
Histopathology of *G. mellonella* infected with *C. krusei* and *C. albicans* and treated with different antifungals. *Galleria mellonella* was infected with 5×10^5^ cells/larva of *C. albicans* SC5314 (**C–H**), or with 5×10^6^ cells/larva of *C. krusei* ATCC 6258 (**K–P**). After 96 hours of infection, larvae were processed for histopathology as described in Material and Methods. (**A, B, I, J**), uninfected controls; (C, D, K and L), untreated controls; (E, F, M and N), larvae treated with fluconazole (12 mg/kg); (**G, H, O and P**), larvae treated with amphotericin B (4 mg/kg). (**A, C, E, G, I, K, M, O**), low magnification; (**B, D, F, H, J, L, N and P**), high magnification.

To complement these studies, we performed histopathology of infected and treated larvae. *Candida albicans* (Figure 8C and 8D) and *C. krusei* (Figure 8K and 8L) were found both in yeast and filament forms. The antifungal treatment with fluconazole (12 mg/kg) in larvae infected with *C. albicans* or *C. krusei* decreased the number of yeasts. Moreover, the fungi were mainly found in defined structures surrounded by *G. mellonella* cells (Figure 8E, F, M, N). Amphotericin B (4 mg/kg) had the same effect as fluconazole, although fewer yeast cells were found with this treatment (Figure 8G, H, O, P). The antifungals did not have a different effect on larvae infected with *C. albicans* or *C. krusei*. Treatment with the antifungals alone did not have any effect on the histopathology of the larvae (result not shown).

### Effects of amphotericin B and fluconazole on the physiology of *G. mellonella* during *C. albicans* and *C. krusei* infection

**Figure 9:**
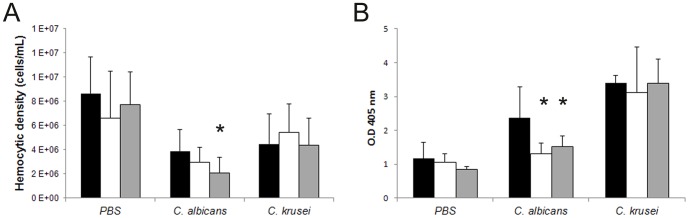
Effect of antifungal treatment of haemocyte density and melanization of *G. mellonella* infected with *C. krusei* or *C. albicans*. (**A**) Hemocytic density of *G. mellonella* infected with *C. albicans* SC5314 or *C. krusei* ATCC 6258 treated with amphotericin B (4 mg/kg) or fluconazole (64 mg/kg). (**B**) Optical Density (OD) of the haemolymph of *G. mellonella* infected with *C. albicans* or *C. krusei* treated with amphotericin B (4 mg/kg) or with fluconazole (64 mg/kg). Black bars, no treatment; grey bars, fluconazole; white bars, amphotericin B. * p<0.05.

Antifungals have immunomodulatory properties in mammals and in *G. mellonella* [54]–[56]. We studied the effect of Amphotericin B (4 mg/kg) and fluconazole (12 and 64 mg/kg) on haemocyte density, melanization and phagocytosis during *G. mellonella* infection by *C. krusei* and *C. albicans*. None of the antifungal treatments influenced the haemocyte density of *C. krusei* infected larvae. However, fluconazole (64 mg/kg) reduced the haemocyte density in larvae infected with *C. albicans* by two fold (p = 0.017, Figure 9A).

None of the antifungals had a significant effect on the melanization of larvae infected with *C. krusei*. In contrast, antifungal treatment of larvae infected with *C. albicans* reduced melanization after 24 hours of infection. Fluconazole (64 mg/kg) and amphotericin B (4 mg/kg) reduced the melanization of these larvae by 1.8 (p = 0.0139) and 1.5 fold, respectively (p = 0.003, Figure 9B). No differences were observed in melanization or phagocytosis after 3 hours of infection with *C. albicans* or *C. krusei*. Antifungal drugs alone did not cause any effect in *G. mellonella* on the parameters analyzed.

### Virulence and antifungal efficacy in *C. elegans* model

**Figure 10:**
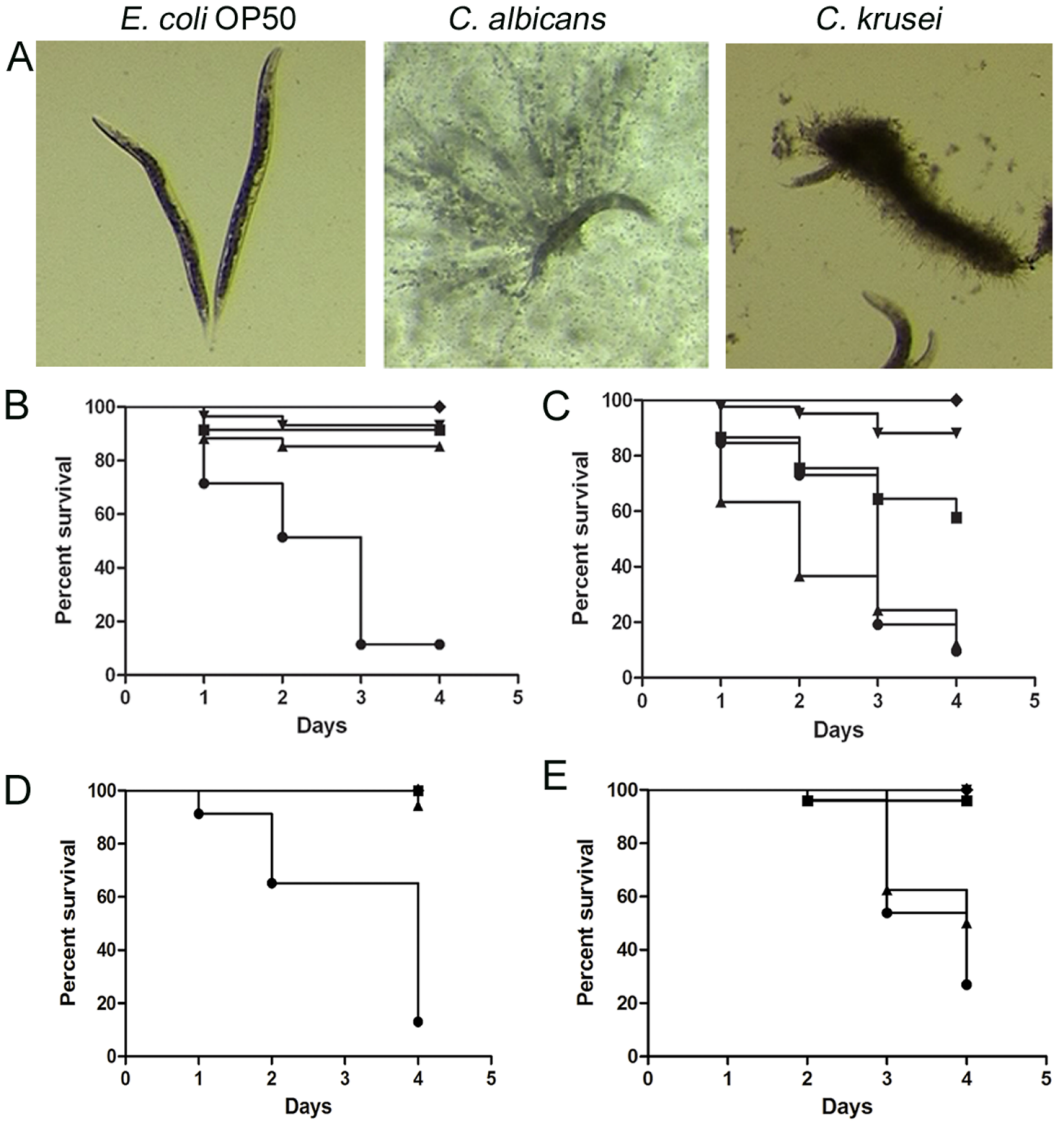
Virulence of *C. krusei* and *C. albicans* in *C. elegans* and antifungal efficacy. *Caenorhabditis elegans* was infected as described in material and methods with *C. krusei* (ATCC 6258), *C. albicans* (SC5314) and *E. coli* OP50. (**A**) Visual appearance of infected worms (50× magnification). (**B**) Antifungal efficacy in *C. elegans* infected with *C. albicans*. ♦ OP50, • *C. albicans*, ▪ *C. albicans* and treated with 2 µg/mL amphotericin B (p<0.0001), ▴ Fluconazole 12 µg/mL (p<0.0001); ▾Caspofungin 2 µg/mL (p<0.0001). (**C**) Antifungal efficacy during *C. krusei* infection ♦ OP50; • *C. krusei*; ▪ *C. krusei* treated with amphotericin B 2 µg/mL; (p<0.0001); ▴Fluconazole 12 µg/mL (p = 0.1207); ▾Caspofungin 2 µg/mL (p<0.0001). (**D**) Effect of voriconazole on survival of *C. elegans* worms infected with *C. albicans* (•, *C. albicans*, ▴, voriconazole 0.25 mg/L (p<0.0001); ▪, voriconazole, 7.5 mg/L (p<0.0001); ▾ voriconazole 10 mg/L (p<0.0001)). (**E**), Efficacy of voriconazole during infection of *C. elegans* by *C. krusei* (•*C. krusei*; ▴voriconazole 0.25 mg/L (p = 0.1217); ▾ voriconazole 7.5 mg/L (9<0.0001); ▪ voriconazole 10 mg/L (p<0.0001)).

The nematode *C. elegans* is another non mammalian model that has been used as a host to study microbial virulence in this study. We also used this model to evaluate the *in vivo* protection of antifungals during *C. krusei* infection such as amphotericin B, fluconazole, voriconazole, caspofungin, and a combination of amphotericin B plus fluconazole. *Candida albicans* and *C. krusei* both killed *C. elegans* worms. In both *Candida* strains, worm death was associated with filamentation of the yeast in the worms (Figure 10A). When we investigated the protection of the different antifungal treatments, we found that all the antifungals protected during *C. albicans* infection at all the concentrations tested (Figure 10B). In contrast, in nematodes infected with *C. krusei*, the behavior of the antifungals was different: amphotericin B only protected at concentrations ≥2 µg/mL and fluconazole was not protective at any of the concentrations used (Figure 10C). Caspofungin showed similar protection as the one observed when the worms were infected with *C. albicans* (Figure 10C). The antifungal combination of fluconazole (12 µg/mL) and amphotericin B (1 µg/mL) did not show any synergistic effect in this model (result not shown). We also studied how voriconazole protected the worms from infection. As shown in Figure 10D, all the concentrations used (0.25, 7.5 and 10 mg/L) protected larvae from infection by *C. albicans*. However, only the higher doses (7.5 and 10 mg/L) showed efficacy during *C. krusei* infection, while the lowest dose (0.25 mg/L) was not protective.

## Discussion

The use of invertebrate hosts to study the virulence of microbial pathogens presents advantages over conventional mammals. Amoebae, nematodes and insect hosts are good models to study virulence and to elucidate host–pathogen interaction. Ethical issues, cost and faster results are other benefits of these models [41], [42], [57]. During evolution, non vertebrate animals have developed immunity against microbial pathogens [42], and for this reason, there are functional and structural similarities between the innate immune system of mammals and insects. So, these models can be used to study immune responses [57].

In this work, we have used two different hosts, *G. mellonella* and *C. elegans*, to investigate virulence of *C. krusei* and antifungal efficacy. Compared to other non-conventional models, *G. mellonella* allows the use of precise pathogen doses by injection, low cost and evaluation of survival at different temperatures. The virulence of five *C. albicans* strains with mutations in genes related to filamentation was evaluated in *G. mellonella* and it was demonstrated that this model is useful as a filamentation assay [53]. In the case of *C. neoformans*, the virulence of different isolates, morphogenesis and antifungal treatments in *G. mellonella* showed good correlation with mammalian system [57], [58]. Previous work demonstrated that *C. elegans* is susceptible to different *Candida* species. For that reason, this host has been used to look for new compounds with antifungal activity [44], [46]. Besides, the available *C. elegans* mutant animals defective in signaling pathways involved in the immune system allows the study of the molecular mechanisms of host-pathogen interaction [59]. However, there are also some cases in which there is no correlation between virulence in mammals and *G. mellonella* [60], so further studies are required to validate the use of these models.

For these reasons, *C. krusei* offers a good model to validate the use of invertebrate models. This yeast shows reduced virulence in mammalian systems and fungal burden is significantly lower in animals infected with *C. krusei* than in animals infected with other fungal pathogens, such as *C. albicans* [61], so it is possible to compare its virulence with other highly virulent yeasts. Moreover, *C. krusei* is intrinsically resistant to fluconazole, so it offers an excellent model to correlate antifungal efficacy *in vitro* and *in vivo*.

Previous work showed that *G. mellonella* infected with 2×10^6^ cells/larva of *C. krusei* killed 20% of the larvae after 72 hrs [62]. In our work, we have reproduced the model and observed that larvae killing can be faster by increasing the pathogen concentration. However, *C. krusei* was less virulent than *C. albicans* because the amount of yeast required to cause 100% death on the fourth day was 10 times higher. This is also in agreement with the reduced virulence of *C. krusei* in mammalian models [63], [64], but also indicates that *G. mellonella* offers a simple method to study virulence traits of *C. krusei*. This finding is of particular interest, since not every microorganism (i.e., *Pneumocystis murina*) can cause disease in *G. mellonella* [65].

The possibility to incubate *G. mellonella* at different temperatures is one of the best advantages of this model because it permits to study virulence at both environment and mammalian temperatures. The virulence of some pathogenic microorganisms (such as *Cryptococcus neoformans*, *Fusarium* spp and *Acinetobacter baumannii*) in *G. mellonella* is affected by the incubation temperature of the larvae after inoculation [66], [67]. In contrast, no statistical difference in the virulence of *C. krusei* was observed between the two temperatures. This correlates with the growth rate of *C. krusei* at both temperatures. In contrast to *C. albicans*, *C. krusei* growth was less affected by the temperature. Interestingly, *G. mellonella* seems to better tolerate environmental temperature than physiological temperature, and it would be expected that immunity is impaired at high temperature. However, our data indicate that in the case of fungi with reduced virulence, immunity at high temperature can control infection, and virulence of the fungus is more dependent on virulence traits of the yeast. *Candida krusei* and *C. albicans* produced filaments in *G. mellonella*, although the morphology was different. *Candida krusei* tended to form cell aggregates with melanization, characteristic of encapsulation. This result indicates that *G. mellonella* differentially recognizes pathogenic yeasts, which can be related to the different virulence exhibited by these two *Candida* species.

Decrease in the amount of haemocytes has been associated with increased susceptibility to fungal infections [39]. *Candida krusei* induced a reduction in the proportion of haemocytes in a similar way as *C. albicans*. This result suggests a mechanism of phagocytosis avoidance by which *Candida* species induce killing of *G. mellonella*, but does not explain the difference in virulence shown by the different *Candida* spp. This reduction might be a consequence of haemocyte explosion after filamentation of these yeasts after internalization. Interestingly, *Cryptococcus neoformans* does not cause a reduction in the number of hemocytes in the first two hours post-infection [58], [66], which might correlate with the fact that this fungus is an intracellular pathogen and can survive in phagocytic cells without affecting their viability [68], [69]. In addition, haemocytes are recruited at infection sites to form clumps or nodules [38], [70], so it is also possible that a proportion of the haemocytes migrate from the haemolymph to the infection sites. In agreement with our findings, it has been described that *C. albicans* induced a reduction in the concentration of haemocytes. In contrast, larvae infected with *S. cerevisiae* showed higher survival and haemocytic concentration [39]. Moreover, the compound [Ag2 (mal) (phen3)] increased the survival of larvae infected with *C. albicans*, and also increased the haemocytic concentration [71]. Phagocytosis of *C. krusei* and *C. albicans* was also lower compared to other fungi, such as *C. neoformans*. There are several mechanisms that could account for this phenomenon. *Candida* spp might be poorly phagocytosed due to impaired pathogen recognition by insect haemocytes. In addition to the reduction of haemocyte density and haemocyte explosion after filament formation discussed above could also explain the reduced phagocytosis observed. The future development of *in vitro* models to study yeast-haemocyte interaction will be of great help to fully characterize these phenomena.

Melanization is a humoral response of the insect that is catalyzed by the enzyme phenoloxidase, and the reaction occurs through the formation of capsules that surround foreign particles [72]. We observed a fast melanization process after infection with *C. krusei*. The degree of melanization depended on the inoculum concentration, but not on the viability of the cells, indicating that melanization is an unspecific process that depends on the presence of foreign particles.

One of the main findings of our work is the correlation between the *in vivo* efficacy of antifungals during *C. albicans* and *C. krusei* infection and their *in vitro* susceptibility profiles. Fluconazole did not have any protective effect during *C. krusei* infection in both *G. mellonella* and *C. elegans* models. Similar findings have been obtained with protection in immunosuppressed mice [63], [73], which validates the use of non-mammalian models to study antifungal efficacy. Due to the simplicity of these models, we believe that these hosts offer suitable and reliable systems to evaluate antifungal efficacy *in vivo*. In this sense, *C. elegans* has been successfully used to perform high-throughput assays to evaluate fungal susceptibility to different types of compounds [46], [74]–[76]. However, more information with resistant strains is required to fully validate their use. We also noticed differences in the protection between *C. albicans* and *C. krusei in vivo* after treatment with voriconazole, amphotericin B and caspofungin. During *C. krusei* infection, the caspofungin and amphotericin B concentrations required for protection were always higher than during *C. albicans* infection. Although *C. krusei* is considered susceptible to the three drugs, it has reduced susceptibility to caspofungin and amphotericin B compared to *C. albicans* [20], [77], [78]. So our data are again in agreement with the different susceptibility profile shown by these species *in vitro*. While several articles suggest molecular mechanisms for the resistance to fluconazole exhibited by *C. krusei*, it is not known why this species is less susceptible to amphotericin B and caspofungin than *C. albicans*. The survival experiments correlated with the fungal burden observed in the larvae. Reduction of the fungal burden was very significant during *C. albicans* with all the antifungals. In contrast, in larvae infected with *C. krusei*, fluconazole had no effect on CFUs and the effect of amphotericin B was less pronounced than in larvae inoculated with *C. albicans*. These data indicate that larvae response is less dynamic during *C. krusei* infection, which poses a limitation to perform pharmacodynamic studies in this infection model. Similar findings have been found in mammalian models. In immunosuppressed mice, fluconazole does not protect during *C. krusei* infection and amphotericin B had a partial effect, while anidulafungin treatment resulted in almost full survival of the animals [79]. In agreement, in another study, fluconazole had a very moderate effect in reducing fungal burden in neutropenic mice compared to other azoles, such as isavuconazole [64]. The use of antifungal combinations has not been sufficiently explored to study the pharmacodynamics response during *C. krusei* infection, and we believe that non mammalian models might offer a simple and easy procedure to evaluate this important issue.

*Caenorhabditis elegans* is also useful to test antifungal efficacy against several pathogenic fungi, including *Candida* spp and *Fusarium* spp [44], [80]. We found very similar results with *C. elegans*, and these results are comparable with the ones found in *G. mellonella*. This finding is important in the context of our work, because we have been able to reproduce very similar results using two different and independent host models. Despite the differences in the immune responses between nematodes and insects, *C. krusei* and *C. albicans* were virulent in both hosts. These results strongly support the use of these hosts as screening models. Interestingly, we could not find significant differences in the virulence of these species in *C. elegans*, in contrast to the results found in *G. mellonella*, where *C. albicans* was more virulent than *C. krusei*. We believe that this difference between the behavior of the different yeast species in *G. melonella* and *C. elegans* is the temperature at which the virulence experiments are performed, which is significantly lower in *C. elegans*.

Understanding fungal pathogenesis and the antifungal discovery are key aspects in medical mycology. Non-conventional models represent an important alternative for *in vivo* studies, even in the case of organisms that present low virulence in mammalian systems, such as *C. krusei*. Our results also demonstrate the feasibility of non-mammalian models to identify new antifungal compounds against resistant species. The correlation of the virulence of pathogenic fungi in mammals and non-mammalian models is still unclear. However, there is increasing evidence that some virulence phenotypes are reproduced in invertebrate models. For this reason, we believe that more studies to validate the full use of these hosts are required in the future.
